# Time course of immature platelet count and its relation to thrombocytopenia and mortality in patients with sepsis

**DOI:** 10.1371/journal.pone.0192064

**Published:** 2018-01-30

**Authors:** Kansuke Koyama, Shinshu Katayama, Tomohiro Muronoi, Ken Tonai, Yuya Goto, Toshitaka Koinuma, Jun Shima, Shin Nunomiya

**Affiliations:** 1 Division of Intensive Care, Department of Anesthesiology & Intensive Care Medicine, Jichi Medical University School of Medicine, Tochigi, Japan; 2 Department of Emergency Medicine, Jichi Medical University School of Medicine, Tochigi, Japan; Royal College of Surgeons in Ireland, IRELAND

## Abstract

**Introduction:**

The pathogenesis of thrombocytopenia in patients with sepsis is not fully understood. The aims of this study were to investigate changes in thrombopoietic activity over time by using absolute immature platelet counts (AIPC) and to examine the impact of platelet production on thrombocytopenia and mortality in patients with sepsis.

**Methods:**

This retrospective observational study included adult patients with sepsis admitted to the intensive care unit at a university hospital. Two hundred five consecutive sepsis patients were stratified into four groups according to nadir platelet count: severe (nadir ≤40×10^3^/μL), moderate (41–80×10^3^/μL), or mild thrombocytopenia (81–120×10^3^/μL), or normal-increased platelet count (>120×10^3^/μL). The development of thrombocytopenia was assessed during the first week; mortality was assessed at day 28.

**Result:**

Of the 205 patients included, 61 (29.8%) developed severe thrombocytopenia. On admission, AIPC did not differ among the four groups. In patients with severe thrombocytopenia, AIPC decreased significantly from days 2 to 7, but remained within or above the normal range in the other three groups (overall group comparison, *P*<0.0001). Multivariate analysis including coagulation biomarkers revealed that AIPC was independently associated with the development of severe thrombocytopenia (day 3 AIPC, odds ratio 0.49 [95% confidence interval (CI) 0.35–0.66], *P*<0.0001; day 5 AIPC, 0.59 [95% CI 0.45–0.75], *P*<0.0001). AIPC was a significant predictor of 28-day mortality in Cox hazard models adjusted for Acute Physiology and Chronic Health Evaluation II and Sequential Organ Failure Assessment scores (day 3 AIPC, hazard ratio 0.70 [95% CI 0.52–0.89], *P* = 0.0029; day 5 AIPC, 0.68 [95% CI 0.49–0.87], *P* = 0.0012).

**Conclusions:**

Thrombopoietic activity was generally maintained in the acute phase of sepsis. However, a decrease in AIPC after admission was independently associated with the development of severe thrombocytopenia and mortality, suggesting the importance of suppressed thrombopoiesis in the pathophysiology of sepsis-induced thrombocytopenia.

## Introduction

Thrombocytopenia is one of the most serious and frequent complications in sepsis [1–3]. More than 50 percent of sepsis patients develop a low platelet count, ranging from a mild decrease to severe thrombocytopenia. Several studies have revealed that thrombocytopenia is a strong predictor of mortality in critically ill patients [4, 5]. In particular, thrombocytopenia is associated with a 1.4–2.1-fold increased risk for mortality in patients with sepsis [1, 3], and the magnitude of platelet reduction is reported to be associated with poor outcome [6]. In very severe thrombocytopenia (platelet count <20×10^3^/μL), the mortality rate increases to about 70% in patients with bloodstream infection [7].

Thrombocytopenia reflects the severity of dysfunction in blood-clotting systems. A low platelet count is one indicator of organ dysfunction in intensive care unit (ICU) scoring systems, such as the Sequential Organ Failure Assessment (SOFA) score [8]. Platelet count is also used in the diagnosis of sepsis-induced coagulopathy and disseminated intravascular coagulation [9, 10]. Furthermore, the clinical significance of platelet count had increased because of recent changes in the definition of sepsis, with organ dysfunction now required for diagnosis [11]. However, the precise mechanism of thrombocytopenia and its association with disease severity and outcome in sepsis remains unclear.

Thrombocytopenia is caused by increased platelet consumption, decreased platelet production, or a combination of both. The mechanisms of thrombocytopenia in sepsis may be multifactorial; however, septic thrombocytopenia arises mainly from increased platelet consumption resulting from platelet activation, adhesion to other immune cells, or thrombus formation [2, 12]. During sepsis, platelets are activated by pathogen-associated molecular patterns and/or inflammatory cytokines, making cell-to-cell contact with other immune cells [13]. Thrombin is a potent platelet agonist, which is generated as an uncontrolled procoagulant response to infection. Excessive thrombin formation and other platelet activation in septic coagulopathy lead to widespread microvascular thrombus and result in platelet consumption [12]. Several studies have confirmed that thrombocytopenia is associated with coagulation derangement in septic patients [14–16]. However, few studies have assessed platelet production and its behavior over time in sepsis-induced thrombocytopenia.

The gold standard for evaluating thrombopoiesis is the analysis of a bone marrow specimen [17, 18]. This invasive procedure, however, is technically difficult to perform on a daily basis and is not often justified under clinical conditions, such as with sepsis. In recent years, non invasive assessment of platelet turnover has been performed by measuring the immature platelet fraction (IPF) [19, 20]. In RNA-binding fluorescence flow cytometry, immature platelets, which are larger and more fluorescent than mature platelets, are gated with a preset algorithm and are measured simultaneously with the complete blood count. This fully automated measurement of IPF is reliable, reproducible, and available in daily clinical practice [21].

IPF is usually reported as the percentage IPF (the percentage of platelets with above-threshold RNA), however it can also be expressed as the absolute immature platelet count (AIPC), which is the actual number of immature platelets per unit volume (%IPF × platelet count). Percentage IPF reflects the balance between platelet production (increase in immature platelet count) and platelet consumption (decrease in total platelet count). A high IPF indicates, for example, recovery of bone marrow function after chemotherapy [22] or peripheral platelet destruction, such as autoimmune thrombocytopenic purpura [21]. In contrast, AIPC is considered to specifically reflect the daily platelet production [23]. The role of AIPC in thrombocytopenia is similar to that of the reticulocyte production index in evaluating anemia: correction for mature platelets is not required because the lifespan of an immature platelet is less than 24 h [24, 25]. Therefore, a low AIPC in thrombocytopenia suggests decreased thrombopoiesis, such as in aplastic conditions, or an inadequate megakaryocytic response [19, 21].

We recently reported that both IPF and AIPC were elevated in patients with sepsis on the day of ICU admission, reflecting increased platelet production and platelet consumption resulting from septic coagulopathy [26]. Other studies have reported that increased IPF predicted infection and the development of sepsis [27, 28]. However, thrombopoietic potential and its changes over time during sepsis have not been determined, especially in patients with severe or prolonged thrombocytopenia.

The aims of this study were to investigate thrombopoietic activity over time in the acute phase of sepsis and to examine the impact of platelet production on thrombocytopenia and mortality in these patients. We also evaluated coagulation biomarkers to compare the coagulation derangement with thrombopoiesis in relation to the development of thrombocytopenia and mortality. We hypothesized that decreased platelet production contributes to the development of severe thrombocytopenia over the course of sepsis, in addition to platelet consumption. To investigate these roles, we stratified patients with thrombocytopenia according to severity and evaluated changes in thrombopoietic activity over time with the AIPC, which allows for non-invasive, repeated measurements on a daily basis.

## Methods

### Study design and settings

This was a single-center, retrospective, observational study conducted at a 14-bed medico-surgical ICU at Jichi Medical University Hospital. Medical records of all patients admitted to the ICU between April 2014 and September 2016 were retrospectively reviewed. Patients older than 18 years who were diagnosed with sepsis on ICU admission were included in the study. Sepsis was defined as an acute change in SOFA score by 2 points or more as a consequence of a proven or suspected infection according to Sepsis-3 criteria [11]. Exclusion criteria were age younger than 18 years; presence of hematologic disorders, including platelet disorders; decompensated liver cirrhosis or failure; history of chemotherapy; and therapeutic anticoagulation or blood transfusion during the preceding 4 weeks. This study was approved by the Institutional Research Ethics Committee of Jichi Medical University, which waived the requirement for informed consent because of the retrospective study design.

Our facility provides 24-h coverage by attending ICU physicians. Patient management followed the Surviving Sepsis Campaign Guideline, with the goal of initial resuscitation and infection control [29]. Patients received mechanical prophylactic treatment and concomitant low-dose heparin, which was administered after confirmation of no active bleeding or severe coagulopathy.

Our institutional criteria for transfusion of platelet concentrate were platelet count <20×10^3^/μL, presence of active bleeding, or invasive procedures as decided by the ICU physicians. Fresh frozen plasma transfusion was also at the discretion of the ICU physicians, and was limited to patients at risk for bleeding or complications.

### Data collection

Descriptive data, including demographic data, diagnosis, sources of infection, and clinical data, were collected from the electronic medical records of all eligible patients. Severity indices, including Acute Physiology and Chronic Health Evaluation (APACHE) II [30] and SOFA scores [8], were calculated on the day of ICU admission. Clinical variables and treatments, such as transfusion of blood products, including red blood cells, fresh frozen plasma, and platelet concentrate, were recorded daily. Clinical outcomes were assessed according to ICU day and all-cause 28-day mortality.

### Measurement of IPF, AIPC, and coagulation biomarkers

Platelet counts, IPF, and coagulation biomarkers are routinely measured on consecutive days in patients with sepsis at our ICU. Peripheral blood samples were collected on ethylene diamine tetra-acetic acid (EDTA) for complete blood count (CBC). Automatic measurement of IPF was performed simultaneously with the CBC using an XE-5000 hematology analyzer (Sysmex, Kobe, Japan). Platelets were stained with RNA-binding fluorescent dyes and passed through a semiconductor diode laser beam in a flow cytometer. According to the fluorescent intensity and forward light scatter measurements, immature platelets were gated with a preset algorithm and counted. IPF corresponds to the fraction (%) of immature platelets in the total platelet population. AIPC was calculated as the absolute number of immature platelets per unit volume (%IPF × platelet count). We recently confirmed the following reference values at our institution: median IPF, 2.1% (range 1.6–3.5%); median AIPC, 4.2×10^3^/μL (range 3.0–6.4×10^3^/μL) [26].

Coagulation biomarkers, including prothrombin time–international normalized ratio (PT-INR), fibrin degradation product, thrombin–antithrombin complex (TAT), and protein C, were assayed with a CS-2100i automatic coagulation analyzer (Sysmex). Berichrom assays (Siemens Healthcare Diagnostics, Tokyo, Japan) were used for protein C; the TAT test F enzyme immunoassay (Sysmex) was used for measurement of TAT levels.

### Data analysis

In this study, thrombocytopenia was defined as a platelet count of 120×10^3^/μL or less, according to our institutional reference values (130–369×10^3^/μL). The lowest platelet count within the first 7 days after ICU admission was used to stratify patients as having severe (≤40×10^3^/μL), moderate (41–80×10^3^/μL), or mild (81–120×10^3^/μL) thrombocytopenia, or a normal-increased platelet count (>120×10^3^/μL). Baseline clinical and presenting characteristics were compared among patients with varying severities of thrombocytopenia.

Differences in clinical characteristics according to nadir platelet count were analyzed with the χ^2^ test for categorical variables. Between-group comparisons of continuous variables were conducted with one-way analysis of variance or the nonparametric Kruskal–Wallis test with/without Steel–Dwass pairwise comparisons for non-normally distributed data.

To evaluate the time course of platelet count and AIPC in the acute phase of sepsis, we unified the study cohort by excluding patients with a baseline platelet count below 80×10^3^/μL on ICU admission. Changes in platelet count, AIPC, and coagulation biomarker concentrations over time in the four groups were compared with multiple analysis of variance.

The relationship between AIPC and the development of severe thrombocytopenia was assessed using logistic regression analysis with unadjusted and adjusted models including coagulation variables. For 28-day mortality, univariate Cox proportional hazards models were used to fit the time from ICU admission to outcome. Cox multivariate models were used to confirm the independent prognostic value of AIPC after adjustment for coagulation biomarkers (model 1) and APACHE II and SOFA scores (model 2). A *P*-value of 0.05 was considered statistically significant. All analyses were conducted with JMP statistical software (ver. 12; SAS Institute, Tokyo, Japan).

## Results

### Baseline clinical characteristics

Two hundred seventy-two patients with sepsis were admitted to the ICU during the study period. Sixty-seven patients were excluded according to exclusion criteria. Data from the remaining 205 patients were included in the study.

Table 1 gives baseline characteristics and outcomes of the study cohort according to nadir platelet count during the first 7 days. Of the 205 patients with sepsis, 61 (29.8%) developed severe thrombocytopenia, 46 (22.4%) developed moderate thrombocytopenia, 44 (21.5%) developed mild thrombocytopenia, and 54 (26.3%) had normal-increased platelet counts.

**Table 1:** Baseline characteristics and outcomes of the 205 patients with sepsis.

	Severe thrombocytopenia (n = 61)	Moderate thrombocytopenia (n = 46)	Mild thrombocytopenia (n = 44)	Normal-increased platelet count (n = 54)	P*
**Demographics**					
Age, years	68.6 ± 14.4	69.7 ± 14.2	70.0 ± 13.2	65.1 ± 15.9	0.29
Male, n (%)	32 (52.5)	31 (67.4)	26 (59.1)	29 (53.7)	0.41
**Source of sepsis**, n (%)					
Pulmonary infection	12 (19.7)	14 (30.4)	13 (29.5)	12 (22.2)	0.57
Abdominal infection	31 (50.8)	22 (47.8)	23 (52.3)	25 (46.3)	0.91
Urinary tract infection	4 (6.6)	1 (2.2)	4 (9.1)	2 (3.7)	0.45
Soft tissue infection	8 (13.1)	5 (10.9)	1 (2.3)	12 (22.2)	0.031
Blood stream infection	1 (1.6)	0 (0.0)	1 (2.3)	0 (0.0)	0.43
Other	5 (8.2)	4 (8.7)	2 (4.5)	3 (5.6)	0.81
**Comorbidities**, n (%)					
IHD	5 (8.2)	7 (15.2)	7 (15.9)	5 (9.3)	0.51
CHF	8 (13.1)	4 (8.7)	8 (18.2)	5 (9.3)	0.49
Arrhythmia	4 (6.6)	6 (13.0)	5 (11.4)	7 (13.0)	0.62
COPD	2 (3.3)	3 (6.5)	4 (9.1)	4 (7.4)	0.26
CKD	15 (24.6)	10 (21.7)	11 (25.0)	11 (20.4)	0.93
CVD	8 (13.1)	7 (15.2)	6 (13.6)	7 (13.0)	0.98
**Severity of illness**					
APACHE II score	29.1 ± 7.3	25.7 ± 8.1	23.2 ± 7.8	22.3 ± 7.6	<0.0001
SOFA score	11 (7–13)	9 (7–10)	8 (5–9.8)	6 (4–7)	<0.0001
**Prognosis**					
ICU days	11 (6.5–18)	9 (6–13.3)	7.5 (4.3–12.5)	7.5 (5–11)	0.0036
28-day mortality, n (%)	16 (26.2)	3 (6.5)	2 (4.6)	1 (1.9)	<0.0001

Data are expressed as mean ± SD, median (interquartile range), or No. (%).

*Comparison among the four groups of patients.

IHD, ischemic heart disease; CHF, chronic heart failure; COPD, chronic obstructive pulmonary disease; CKD, chronic kidney disease; CVD, cerebrovascular disease; APACHE, acute physiology and chronic health evaluation; SOFA, sequential organ failure assessment.

The primary source of infection was largely similar among the groups, with a few exceptions. Patients with normal-increased platelet counts were more often admitted with sepsis due to soft tissue infection. Patients with severe thrombocytopenia were more severely ill on ICU admission, as reflected by higher APACHE II and SOFA scores than the other three groups. Patients with severe thrombocytopenia had significantly higher mortality rates (26.2%) than patients with mild or moderate thrombocytopenia or normal-increased platelet counts (6.5, 4.6, and 1.9%, respectively). Patients with severe thrombocytopenia were significantly more likely to be transfused with platelet concentrate (severe, 21 [34.4%]; moderate, 3 [6.5%]; mild, 0 [0%]; normal-increased, 0 [0%]; *P*<0.0001) and with fresh frozen plasma (severe, 12 [19.7%]; moderate, 2 [4.4%]; mild, 1 [2.3%]; normal-increased, 1 [1.9%]; *P* = 0.0012). There were no differences among the groups in rate of red blood cell transfusion (severe, 13 [21.3%]; moderate, 5 [10.9%]; mild, 5 [11.4%]; normal-increased, 9 [16.7%]; *P* = 0.588).

### Time course of platelet count and AIPC during the study period

To evaluate platelet count and AIPC over time, we excluded patients with a baseline platelet count below 80×10^3^/μL on ICU admission. The analysis included 48 patients with severe thrombocytopenia, 43 patients with moderate thrombocytopenia, 44 patients with mild thrombocytopenia, and 54 patients with normal-increased platelet counts.

**Fig 1:**
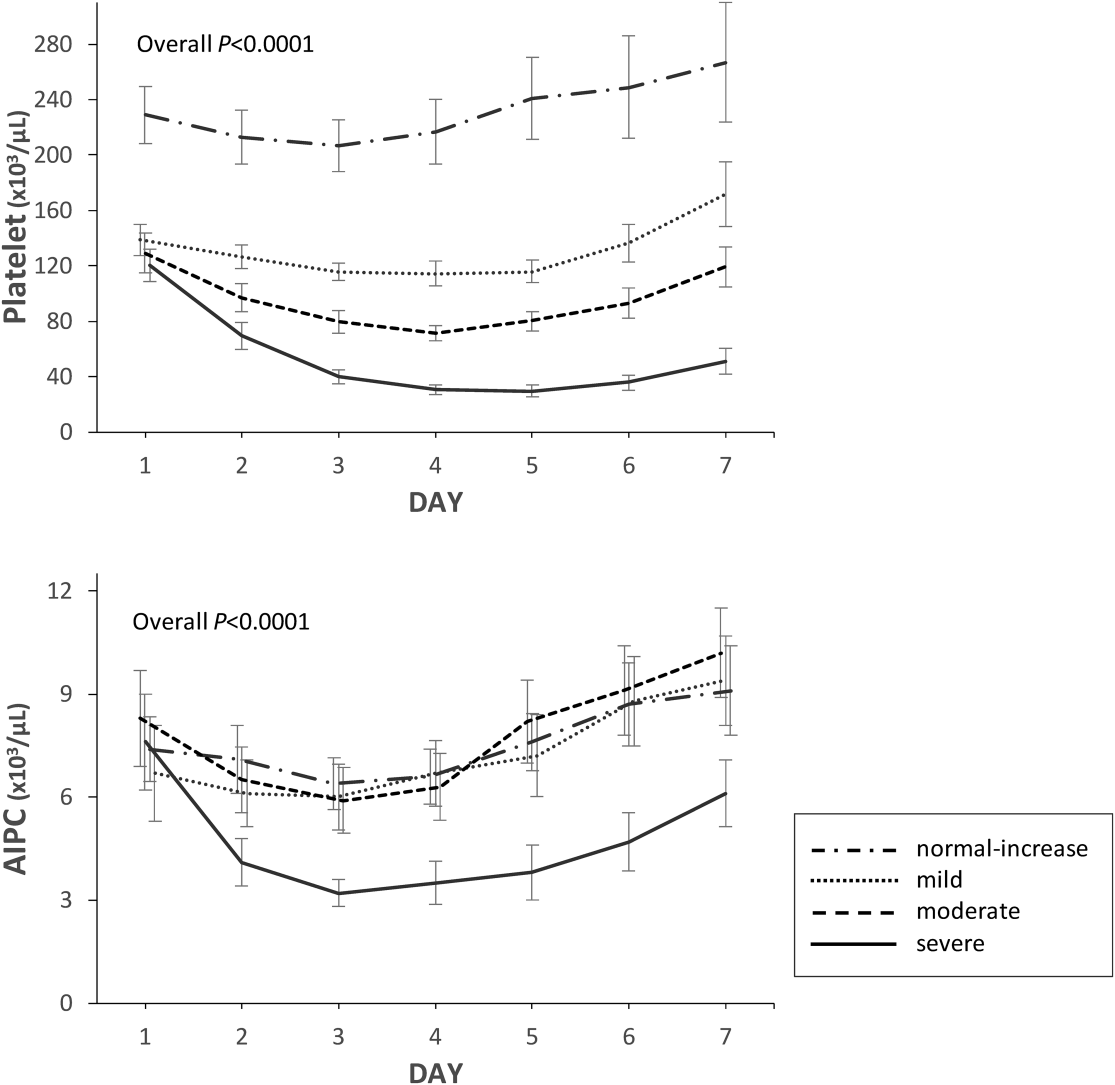
Time course of platelet count and absolute immature platelet count (AIPC). Platelet count and AIPC were measured from admission (day 1) to day 7 in patients with sepsis, classified into four groups according to nadir platelet count during the 7 days: severe thrombocytopenia (nadir platelet count ≤40×10^3^/μL); moderate thrombocytopenia (41–80×10^3^/μL); mild thrombocytopenia (81–120×10^3^/μL); and normal-increased platelet count (>120×10^3^/μL). Data are expressed as mean with the 95% confidence interval shown by the error bars. Multiple analysis of variance was among the four groups.

**Fig 2:**
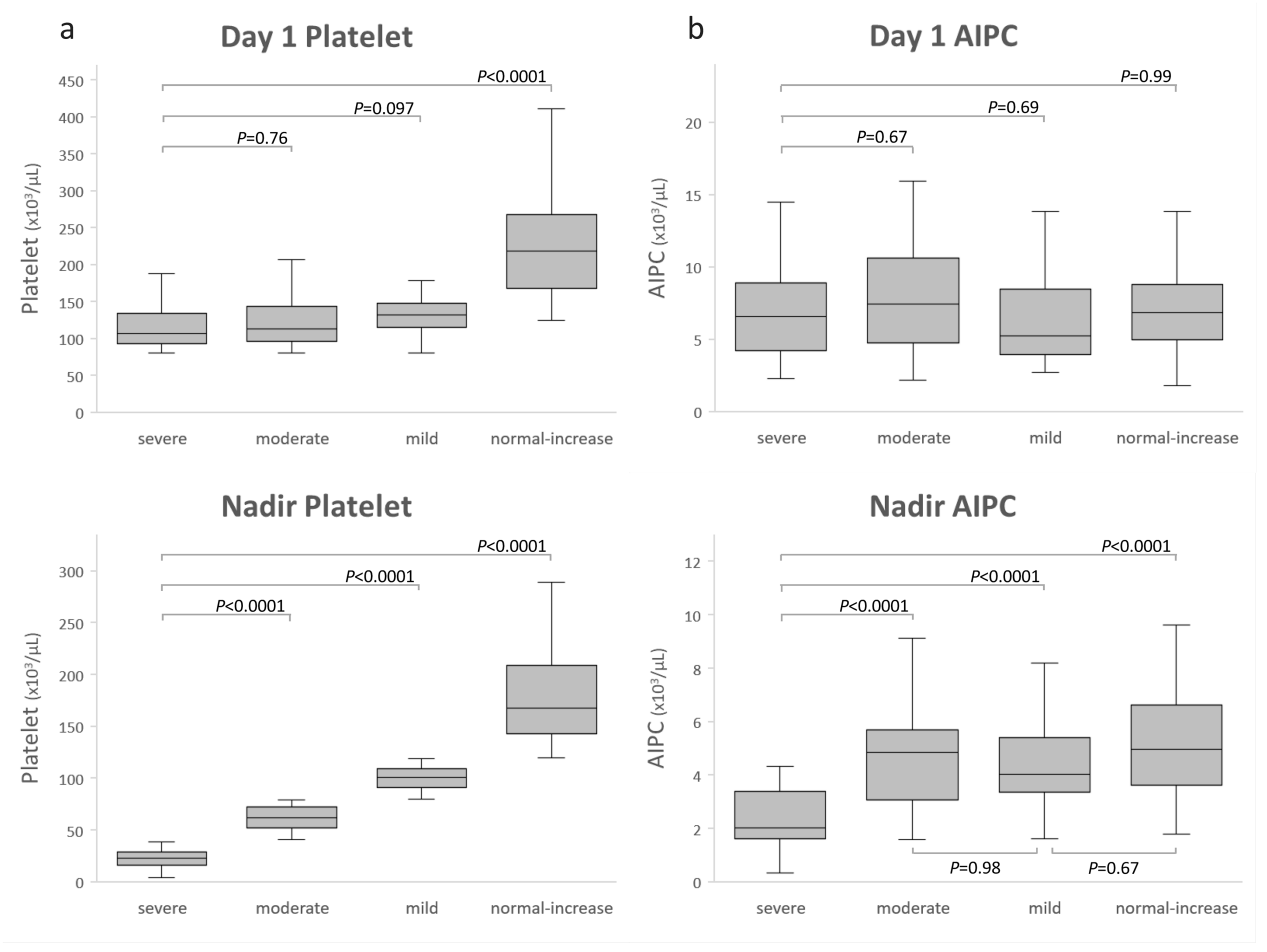
Box plot showing (a) platelet and (b) absolute immature platelet count (AIPC). Platelet count and AIPC on the day of admission (day 1) and at the nadir during the 7 days are shown. Septic patients were classified into four groups according to nadir platelet count during the first 7 days of their ICU stay: severe thrombocytopenia (nadir platelet count ≤40×10^3^/μL); moderate thrombocytopenia (41–80×10^3^/μL); mild thrombocytopenia (81–120×10^3^/μL); and normal-increased platelet counts (>120×10^3^/μL). Multiple pairwise comparison used the Steel–Dwass test.

Changes in platelet count and AIPC from days 1 to 7 according to group are shown in Fig 1. Overall differences in the time course of platelet count and AIPC were significant among the groups (*P*<0.0001). Fig 2 shows the results of multiple comparisons on day 1 and at nadir for platelets and AIPC among groups.

The platelet count and AIPC on day 1 were not significantly different among patients with mild, moderate, and severe thrombocytopenia (Figs 1 and 2). Platelet counts dropped until days 4–5 in these patients. The AIPC in patients with mild or moderate thrombocytopenia and with normal-increased platelet counts were within or above the reference range over the 7 days, suggesting thrombocytopenia was caused by platelet consumption. In contrast, the AIPC in patients with severe thrombocytopenia fell rapidly until day 3 and did not recover to the levels of the other three groups (Fig 1). Nadir AIPC in the severe thrombocytopenia group was significantly lower than those in the other groups, indicating an additional mechanism of suppressed thrombopoiesis might be complicated in these patients.

### Time course of coagulation biomarkers during the study period

**Fig 3:**
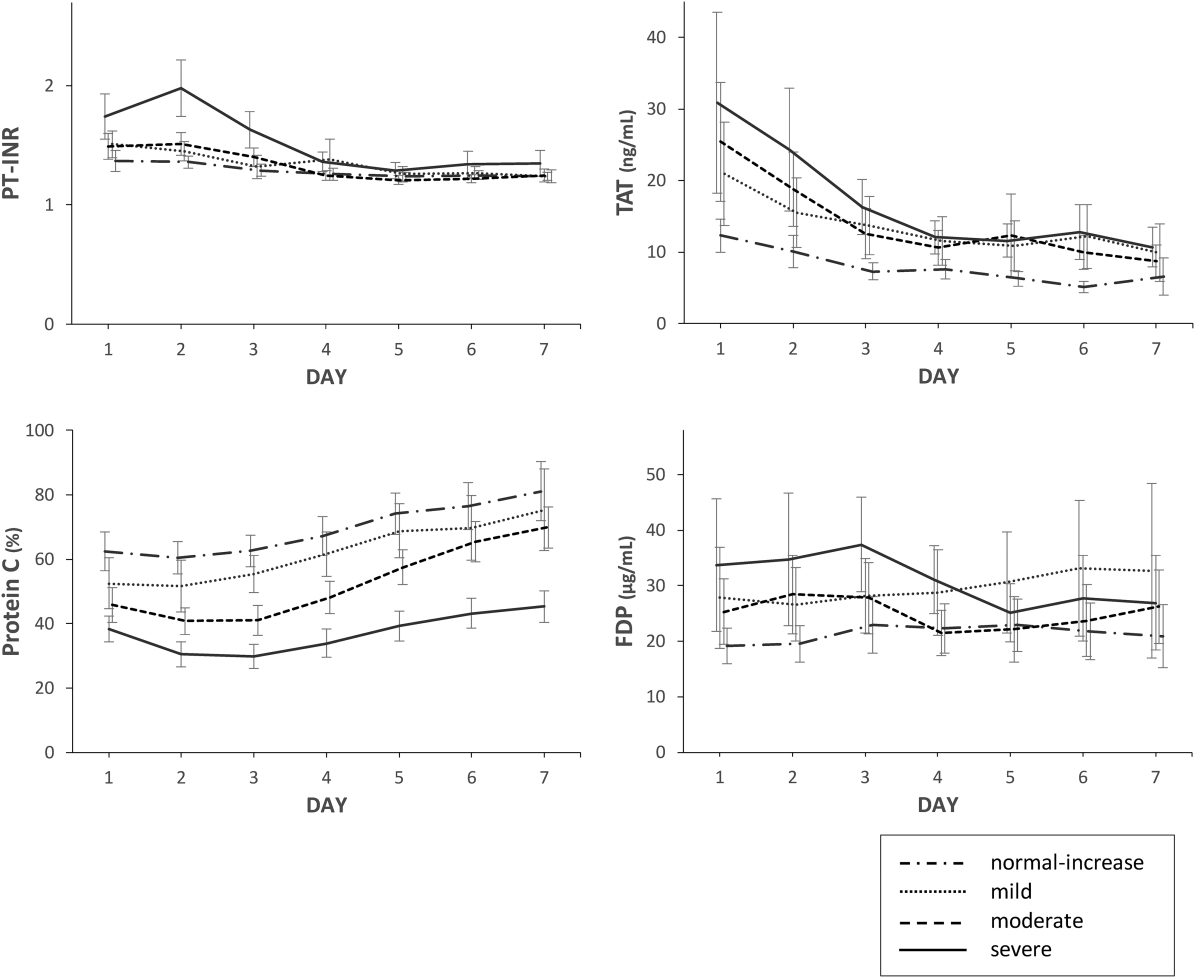
Time course of coagulation biomarkers from admission (day 1) to day 7 in patients with sepsis. Septic patients were classified into four groups according to nadir platelet count during the first 7 days of their ICU stay: severe thrombocytopenia (nadir platelet count ≤40×10^3^/μL); moderate thrombocytopenia (41–80×10^3^/μL); mild thrombocytopenia (81–120×10^3^/μL); and normal-increased platelet counts (>120×10^3^/μL). Data are expressed as mean with the 95% confidence interval shown by the error bars.

To evaluate changes in the severity of sepsis-induced coagulopathy, which is an important cause of thrombocytopenia, we measured coagulation biomarkers over time during the first 7 days of intensive care (Fig 3). On the day of ICU admission, PT-INR and TAT were higher and protein C was lower in patients with severe thrombocytopenia compared with patients with normal-increased platelet counts. These findings may indicate the involvement of consumption coagulopathy, increased thrombin synthesis, and endothelial dysfunction. From days 2 to 5, PT-INR and TAT in patients with severe thrombocytopenia improved to levels of patients with normal-increased platelet counts. These improvements were observed before the platelet count increased, suggesting septic coagulopathy was subsiding.

### AIPC and the development of severe thrombocytopenia

To evaluate the impacts of platelet production and coagulation derangement on the development of severe thrombocytopenia, we conducted univariate and multivariate logistic regression analysis, including AIPC and coagulation biomarkers as variables, at three time points: days 1, 3, and 5 (Table 2). Although AIPC on day 1 was not associated with severe thrombocytopenia, decreased AIPC on days 3 and 5 were significantly associated with the development of severe thrombocytopenia in the univariate logistic regression model (day 3 AIPC, odds ratio [OR] 0.47 [95% confidence interval (CI) 0.35–0.60], *P*<0.0001; day 5 AIPC, OR 0.56 [95% CI 0.45–0.68], *P*<0.0001). We then estimated the independent association between AIPC and the development of severe thrombocytopenia with multivariate logistic regression, adjusting for coagulation biomarkers, which are indicators of platelet consumption resulting from coagulopathy. Even after adjustment, AIPC on days 3 and 5 remained significantly associated with the development of severe thrombocytopenia (day 3 AIPC, OR 0.49 [95% CI 0.35–0.66], *P*<0.0001; day 5 AIPC, OR 0.59 [95% CI 0.45–0.75], *P*<0.0001), suggesting that decreased platelet production independently contributes to the development of severe thrombocytopenia.

**Table 2:** Univariate/multivariate logistic regression models for severe thrombocytopenia development (nadir platelet count <40×10^3^/μL).

Time	Biomarker	Univariate		Multivariate	
		OR (95% CI)	*P*-value	OR (95% CI)	*P*-value
Day 1	AIPC	0.99 (0.92–1.08)	0.98		
	PT-INR	3.6 (1.8–8.6)	0.0003	1.5 (0.65–3.7)	0.35
	FDP	1.01 (1.00–1.02)	0.044	1.01 (0.99–1.02)	0.29
	TAT	1.01 (1.00–1.03)	0.019	1.01 (0.99–1.02)	0.24
	Protein C	0.95 (0.93–0.97)	<0.0001	0.96 (0.93–0.98)	0.0016
Day 3	AIPC	0.47 (0.35–0.60)	<0.0001	0.49 (0.35–0.66)	<0.0001
	PT-INR	9.6 (3.3–34.3)	<0.0001	2.2 (0.46–11.6)	0.31
	FDP	1.02 (1.01–1.03)	0.0058	1.00 (0.98–1.03)	0.79
	TAT	1.04 (1.01–1.07)	0.0065	1.03 (0.98–1.08)	0.18
	Protein C	0.90 (0.87–0.93)	<0.0001	0.93 (0.88–0.96)	<0.0001
Day 5	AIPC	0.56 (0.45–0.68)	<0.0001	0.59 (0.45–0.75)	<0.0001
	PT-INR	1.4 (0.64–3.6)	0.37		
	FDP	1.00 (0.98–1.02)	0.96		
	TAT	1.01 (0.98–1.05)	0.37		
	Protein C	0.91 (0.88–0.94)	<0.0001	0.91 (0.87–0.95)	<0.0001

OR, odds ratio; CI, confidence interval; AIPC, absolute immature platelet count; PT-INR, prothrombin time–international normalized ratio; FDP, fibrin degradation products; TAT, thrombin–antithrombin complex.

### AIPC and mortality

We evaluated the prognostic value of AIPC on day 28 after admission. In univariate Cox proportional hazards models (Table 3), AIPC on days 3 and 5 were associated with 28-day mortality (day 3 AIPC, hazard ratio [HR] 0.65 [95% CI 0.48–0.83], *P* = 0.0001; day 5 AIPC, HR 0.72 [95% CI 0.57–0.88], *P* = 0.0006). After adjustment for coagulation derangement, AIPC on days 3 and 5 remained independently associated with outcome at 28 days (model 1: day 3 AIPC, HR 0.65 [95% CI 0.47–0.85], *P* = 0.0008; day 5 AIPC, HR 0.68 [95% CI 0.49–0.87], *P* = 0.0012). When APACHE II and SOFA scores were included in this model, AIPC on days 3 and 5 remained independent predictors of 28-day mortality (model 2: day 3 AIPC, HR 0.70 [95% CI 0.52–0.89], *P* = 0.0029; day 5 AIPC, HR 0.68 [95% CI 0.49–0.87], *P* = 0.0012).

**Table 3:** Univariate and multivariate Cox regression models to predict 28-day mortality.

Time	Biomarker	Univariate		Multivariate (model 1)		Multivariate (model 2) *	
		HR (95% CI)	*P*-value	HR (95% CI)	*P*-value	HR (95% CI)	*P*-value
Day 1	AIPC	0.96 (0.89–1.05)	0.32				
	PT-INR	2.2 (1.2–3.5)	0.012	2.1 (1.1–3.5)	0.031	1.6 (0.69–3.1)	0.24
	FDP	1.01 (0.99–1.02)	0.32				
	TAT	1.01 (1.00–1.02)	0.0043	1.01 (1.00–1.02)	0.011	1.01 (1.00–1.02)	0.031
	Protein C	0.99 (0.96–1.01)	0.25				
Day 3	AIPC	0.65 (0.48–0.83)	0.0001	0.65 (0.47–0.85)	0.0008	0.70 (0.52–0.89)	0.0029
	PT-INR	6.7 (3.5–11.9)	<0.0001	5.8 (2.0–16.5)	0.0014	4.1 (1.5–11.4)	0.0071
	FDP	1.01 (0.99–1.02)	0.21				
	TAT	1.06 (1.03–1.08)	<0.0001	1.02 (0.99–1.05)	0.10	1.03 (1.00–1.06)	0.049
	Protein C	0.95 (0.92–0.98)	0.0004	0.99 (0.97–1.03)	0.97	1.01 (0.98–1.05)	0.51
Day 5	AIPC	0.72 (0.57–0.88)	0.0006	0.68 (0.49–0.87)	0.0012	0.68 (0.49–0.87)	0.0012
	PT-INR	1.8 (1.2–2.5)	0.012	2.4 (1.3–4.6)	0.013	2.4 (1.2–4.5)	0.015
	FDP	1.02 (1.01–1.04)	0.0082	1.02 (0.99–1.03)	0.086	1.02 (0.99–1.04)	0.13
	TAT	1.02 (0.98–1.04)	0.19				
	Protein C	0.97 (0.94–0.99)	0.0059	0.99 (0.97–1.02)	0.79	1.00 (0.97–1.03)	0.82

HR, hazard ratio; AIPC, absolute immature platelet count; PT-INR, prothrombin time–international normalized ratio; FDP, fibrin degradation products; TAT, thrombin–antithrombin complex.

*The covariates of APACHE II and SOFA scores were considered in multivariate model 2.

## Discussion

In this observational study, we evaluated changes in thrombopoiesis in the acute phase of sepsis by assessing repeated AIPC measurements on consecutive days. We found that the AIPC was increased or in the upper normal range in patients with mild or moderate thrombocytopenia and in those with normal-increased platelet counts, indicating that platelet production was not suppressed in these patients. In patients with severe thrombocytopenia, however, nadir AIPC was significantly lower than in the other groups, suggesting that decreased thrombopoiesis occurred in the course of sepsis. In addition, decreased AIPC was independently associated with the development of severe thrombocytopenia and was a predictor of 28-day mortality in patients with sepsis.

Platelet production is a major mechanism in differentiating the pathophysiology of thrombocytopenia; however, thrombopoiesis in sepsis-induced thrombocytopenia is poorly understood [2]. Previous studies have reported that thrombopoietin, a potent stimulator of thrombopoiesis, increased during septic episodes [31, 32], but the effect of this increase on platelet production was unclear. In neonatal sepsis, Eissa et al. found that the reticulated platelet count was low to normal on the day of ICU admission, whereas the reticulated platelet percentage and thrombopoietin level were increased [33]. They concluded that thrombocytopenia on admission mostly resulted from platelet consumption, and that thrombopoiesis might respond to increased thrombopoietin. In contrast, Cremer et al. studied neonatal sepsis from before diagnosis to days 8–12 and found that AIPC was low during thrombocytopenia, indicative of decreased megakaryopoiesis in sepsis and necrotizing enterocolitis [34]. That study also found that AIPC decreased significantly when the platelet count was lower than 50×10^3^/μL, which is consistent with our results.

In the current study, AIPC was already high in all four groups at the time of ICU admission, which is consistent with other studies [27, 28] and with our previously reported results [26]. This increase in AIPC on admission reflects increased thrombopoiesis in sepsis, possibly resulting from inflammatory cytokines, including thrombopoietin. Therefore, mild and moderate thrombocytopenia might result from platelet consumption, which is partly attributable to septic coagulopathy, demonstrated by increased PT-INR, TAT, and decreased protein C activity in the same period. However, AIPC decreased significantly from day 2 in patients with severe thrombocytopenia compared with other groups. Given that immature platelets are produced and progress to maturity within 24 hours, the low AIPC observed in severe thrombocytopenia could not be explained solely by platelet consumption. In addition, the recovery of AIPC in patients with severe thrombocytopenia was slow and AIPC did not reach the levels of the other groups within the first 7 days. If daily platelet production was not suppressed, AIPC in severe thrombocytopenia would be expected to rapidly return to the levels observed in mild or moderate thrombocytopenia as platelet consumption subsides. Furthermore, a decrease in AIPC was independently associated with severe thrombocytopenia, whereas only protein C activity remained significant until day 5 among biomarkers of coagulopathy in both univariate and multivariate analysis. Given these considerations, the low AIPC observed in this study indicates that suppressed thrombopoiesis additionally contributes to the development of severe thrombocytopenia in patients with sepsis.

Interestingly, changes in AIPC over 7 days were not significantly different among patients with normal-increased platelet counts and those with mild or moderate thrombocytopenia, in whom more increased platelet production is expected. One possible explanation for this finding is that thrombopoiesis decreases slightly in patients with mild and moderate thrombocytopenia, perhaps by the same mechanism involved in patients with severe thrombocytopenia. Another possibility is that it reflects the latency of the increased thrombopoietic response to platelet consumption. Regulators of thrombopoiesis, such as thrombopoietin, tumor necrosis factor-α, and interleukin (IL)-6, all increase in patients with sepsis and correlate with sepsis severity [32, 35]. Tumor necrosis factor-α and IL-6 are secreted as initial proinflammatory cytokines in sepsis, stimulating the production of thrombopoietin [36, 37]. Elevated thrombopoietin levels have been reported in healthy volunteers after endotoxin infusion [38] as well as in children [33] and adults with sepsis [32]. However, no studies except those concerning neonatal sepsis have evaluated the relationship between thrombopoietin and reticulated platelet count, and it is not clear how effectively thrombopoietin increases the production of platelets in the acute phase of sepsis. In an animal model, Kaser et al. found that plasma thrombopoietin levels increased significantly on day 3 and peaked on day 9 after administration of IL-6 [39]. Cerutti et al. reported that elevation of thrombopoietin continued for 11 days before the platelet count started to increase in postoperative patients [40]. Therefore, it might take a few days for the bone marrow to increase thrombopoiesis in response to thrombopoietin and other regulators.

The mechanisms behind suppressed platelet production in septic thrombocytopenia were not determined in the current study. Thrombopoiesis is a complex process featuring various steps, including thrombopoietic stimulus (responsiveness to thrombopoietin), megakaryocyte growth, and release of immature platelets [17]. Thrombopoietin and other stimulators increase in patients with sepsis. Thus, possible mechanisms of suppressed production include decreased responsiveness to thrombopoietin, impaired megakaryocyte function, decreased megakaryocyte count, or destruction of newly formed platelets in the bone marrow. Thiolliere et al. evaluated bone marrow aspirates of 238 ICU patients with thrombocytopenia, including 182 patients with sepsis and septic shock [18]. They found that 221 (92.9%) patients with thrombocytopenia had no megakaryocyte depletion. More than half of these patients (61.3%) had normal marrow, and some (8.4%) showed phagocytic histiocytes.

Hemophagocytic lymphohistiocytosis (HLH), in which severe infection or systemic inflammatory conditions induce excessive activation of macrophages, could be the etiology of severe thrombocytopenia in our study. However, Buyse et al. reported that among 5027 ICU patients, only 75 (1.5%) were diagnosed with HLH; the underlying diseases in these patients were mainly malignancies (76.8%), with infection a less common cause of HLH (23.3%) [41].

In contrast, the autopsy study of Inai et al. found that 13 of 18 patients with sepsis (72.2%) had bone marrow findings of histiocytic hyperplasia with hemophagocytosis (HHH), which was associated with increased levels of IL-6 and IL-8 [42]. Although patients with HHH were more thrombocytopenic than those without HHH, none of the patients with HHH fulfilled the diagnostic criteria for HLH, suggesting that the two are different entities. In the current study, we examined bone marrow from two patients with severe thrombocytopenia and found mild hypocellular marrow with hemophagocytosis (data not shown). Further studies are needed to investigate the mechanism behind decreased thrombopoiesis in patients with sepsis.

In this study, decreased AIPC was independently associated with 28-day mortality, suggesting that thrombopoietic dysfunction itself reflects the severity of illness in septic patients. Evidence suggests that thrombocytopenia is associated with increased mortality in critically ill patients, regardless of underlying disease [4, 5]. However, whether the mechanism of this association is platelet production or consumption remains unclear. In sepsis, several studies have reported an association between sepsis-induced coagulopathy and poor prognosis [43, 44]. We previously reported an association between coagulopathy-related platelet consumption on the day of ICU admission and mortality in patients with sepsis [26]. In the current study, AIPC on admission was normal to increased, and was not associated with mortality. However, on and after day 3, decreased AIPC became a significant predictor of 28-day mortality as septic coagulopathy subsided. These results indicate that the effect the cause of thrombocytopenia has on prognosis depends on the phase and severity, and that each mechanism, platelet production and consumption, is independently associated with mortality.

This study has some potential limitations. First, it was a retrospective, observational study conducted at a single center with a relatively small population size. A large, prospective study is needed to validate our results. Second, we excluded patients with hematologic disorders, chronic liver failure, or a history of myelosuppressive therapy to eliminate possible causes of bone marrow dysfunction other than sepsis. In addition, admission AIPC was not low and the reticulocyte count in peripheral blood was within the normal range (data not shown), suggesting no evidence of bone marrow dysfunction or decreased thrombopoiesis, at least on ICU admission. Further research is needed to validate the clinical utility of AIPC in these patient subgroups. Third, although our management of sepsis followed the Surviving Sepsis Campaign Guideline and did not deviate from standard care, prophylactic anticoagulation and interventions such as platelet concentrate transfusion may have influenced the levels of coagulation biomarkers and AIPC, except at baseline. However, Bat et al. reported that AIPC was not affected by platelet concentrate transfusion and is therefore a reliable marker of bone marrow production [45]. In addition, our institutional indication for platelet transfusion was platelet count <20×10^3^/μL. The impact of transfusion might therefore be small in this study. Last, we did not examine megakaryocytes in bone marrow and did not measure serum thrombopoietin, despite the fact that both are key factors regulating thrombopoiesis. Further studies are needed to determine the processes of impaired thrombopoiesis; this information may lead to the development of new therapeutic targets in sepsis-induced thrombocytopenia.

## Conclusions

Thrombopoietic activity was generally maintained in the acute phase of sepsis, suggesting that platelet consumption could be the main mechanism of thrombocytopenia in septic patients. However, a subsequent decrease in AIPC was associated with the development of severe thrombocytopenia, suggesting the additional mechanism of suppressed platelet production. Furthermore, lower AIPC was associated with mortality, independently of clinical risk as determined by APACHE II and SOFA scores. These preliminary findings indicate that decreased thrombopoiesis may be of clinical importance in assessing septic thrombocytopenia and for risk stratification in patients with sepsis. As a simple and readily available cellular marker, serial measurement of AIPC may promote understanding and determination of the underlying pathophysiology in sepsis-induced thrombocytopenia.

## Supporting information

S1 File Original dataset. (XLSX)

## Abbreviations

AIPC: absolute immature platelet count
APACHE: acute physiology and chronic health evaluation
CI: confidence interval
FDP: fibrin degradation product
HHH: histiocytic hyperplasia with hemophagocytosis
HLH: hemophagocytic lymphohistiocytosis
IL: interleukin
IPF: immature platelet fraction
PT-INR: prothrombin time–international normalized ratio
SOFA: sequential organ failure assessment
TAT: thrombin–antithrombin complex
